# Role of Interventional Oncology in the Management of Neuroendocrine Tumors: 2026 Update

**DOI:** 10.1007/s11912-026-01822-2

**Published:** 2026-08-27

**Authors:** Daniel M. DePietro, Michael C. Soulen

**Affiliations:** Division of Interventional Radiology, Department of Radiology, Perelman School of Medicine, University of Pennsylvania, 1 Silverstein, 3400 Spruce Street, Philadelphia, PA 19104 USA

**Keywords:** Neuroendocrine tumor liver metastases, Liver-directed therapy, Chemoembolization, Radioembolization

## Abstract

**Purpose of Review:**

Summarize recent advances in interventional oncology for the management of neuroendocrine tumor liver metastases (NETLMs), with a focus on evolving evidence for embolization and ablation, integration with systemic therapies, and emerging approaches.

**Recent Findings:**

Recent studies have strengthened the role of liver-directed therapies within multidisciplinary NET care. Long-term data supports transarterial chemoembolization as an effective and repeatable line of therapy. Improved dosimetry techniques are refining radioembolization's role in NETLM treatment, improving outcomes while limiting long-term toxicities. Synergistic treatment combinations have demonstrated promising efficacy. New evidence for focal therapies, including radiation segmentectomy and histotripsy, may expand options for solitary and oligometastatic disease. Concurrent biopsy and molecular profiling increasingly inform treatment decisions.

**Summary:**

Interventional radiologists play an increasingly important role in NETLM management by providing tailored and targeted liver-directed therapies, improving patient outcomes, delaying the start of systemic therapies, and integrating with NET treatment plans. Ongoing studies will further define optimal treatment sequencing and patient selection while expanding interventional treatment options.

## Introduction

Neuroendocrine tumors (NETs) are a heterogenous group of malignancies arising from neuroendocrine cells primarily within the gastroenteropancreatic system and the lung. Up to 80% of NET patients develop liver metastases (NETLMs), which may cause hormonal syndromes, bulk-related symptoms, and decreased quality of life [1–3]. NETLMs are associated with a worse prognosis and hepatic tumor burden independently predicts survival, making their treatment critical to improving outcomes [4–6]. While surgical resection remains the only potentially curative treatment, most patients are not candidates due to multifocal or extensive disease [7]. In such patients, liver-directed therapies (LDTs) play an important role in tumor debulking, symptom control, and delaying progression.

Outcomes for patients with NETLMs have improved in recent years related to development of better therapeutic options and multidisciplinary care [8–14]. With the median survival for a newly diagnosed patient now exceeding 15 years, patients undergo many lines of therapy over their prolonged disease courses. Management increasingly emphasizes treatment sequencing and integration of multimodal therapies.

Interventional radiologists (IRs) contribute to this process through selection and timing of transarterial embolization, ablation, and other interventions. Therefore, IRs must remain familiar with evolving evidence for liver-directed therapies, systemic treatment options, and guideline recommendations to optimize patient outcomes. Rather than provide a comprehensive historical overview of LDTs, as can be found elsewhere, this article highlights recent developments that are reshaping the role of IRs and liver-direct therapy in the management of NETLMs and highlights areas where further research would provide impactful benefit [15–17].

### Current Systemic Therapies, Consensus Guidelines, and Treatment Decisions

While a complete review of current systemic therapies is beyond the scope of this article, a brief overview is provided, with landmark studies summarized in Table 1. Somatostatin analogs (SSAs) are monthly injections that provide carcinoid symptom control and inhibition of tumor growth, typically prescribed as first-line treatment for SSTR-positive tumors [18, 19, 21]. Peptide receptor radionuclide therapy (PRRT) utilizes lutetium-177, a beta-emitting radionuclide, which is chelated to a somatostatin analog to target SSTR-positive tumors. The NETTER-1 trial provided evidence for its use in grade 1–2 midgut NETs after progression on an SSA [9]. The recent NETTER-2 trial demonstrated the efficacy of PRRT as first-line therapy in those with grade 2–3 gastroenteropancreatic NETs (GEP-NETs), the implications of which are discussed later in this review [10]. Multiple small molecule inhibitors are in use, including everolimus and sunitinib. These are typically considered more tumoristatic than tumoricidal, with modest response rates [8, 20, 22]. More recently, cabozantinib, a multi-kinase inhibitor, was evaluated in the CABINET trial, which demonstrated promising results in the later-line treatment of both metastatic pancreatic NETs (pNETs) and extra-pancreatic NETs [12]. This trial is also reviewed in more detail later. Few chemotherapeutic regimens have proven effective in the treatment of NETs outside of the combination of capecitabine and temozolomide (CAPTEM), although this has proven efficacious only in those with pNETs, demonstrating a 40% objective response rate (ORR) in this population, with effective symptom control and cytoreductive capabilities [11, 23, 24]. Other cytotoxic chemotherapies, such as FOLFOX and carboplatin/etoposide, have limited evidence, but may be used in high-grade disease when other options have been exhausted or aggressive features are present (24).

**Table 1:** Landmark clinical trials for systemic therapies in the treatment of neuroendocrine tumors

Clinical trial	Year	Intervention	Study population; *n*	Primary endpoint; results	Other key results	Impact
Promid [18]	2009	Octreotide LAR vs. placebo	Metastatic well-differentiated midgut NETs; *n* = 85	TTP; 14 vs. 6 months (HR 0.34, 95% CI 0.2–0.59, *p* < 0.01)	Benefit seen in functional and nonfunctional tumors; greatest effect with lower NETLM burden and resected primary	First RCT demonstrating antiproliferative effect of SSAs; established SSA use in midgut NETs
Clarinet [19]	2014	Lanreotide vs. placebo	Non-functional metastatic well-differentiated GEP- NETs; *n* = 204	PFS; median not reached vs. 18 months (HR 0.47, 95% CI 0.3–0.73, *p* < 0.01); 24 month PFS 65% vs. 33%	Open label extension showed median PFS of 39 months with continued lanreotide	Extended SSA antiproliferative evidence to pancreatic and hindgut NETs; FDA approval of lanreotide
RADIANT-3 [20]	2011	Everolimus vs. placebo	Progressive pancreatic NETs	PFS; 11 vs. 5 months (HR 0.35, 95% CI 0.27–0.45, *p* < 0.01); 65% risk reduction	64% of everolimus patients had tumor shrinkage vs. 21% placebo, final OS 44 vs. 38 months (*p* = 0.3)	FDA approval of everolimus for progressive pNETs; first targeted therapy approved for pNETs
RADIANT-4 [8]	2016	Everolimus vs. placebo	Progressive non-functional GI or lung NETs	PFS; 11 vs. 4 months (HR 0.48, 95% CI 0.35–0.67, *p* < 0.01); 52% risk reduction	Low ORR (2%) but meaningful stabilization of disease	Expanded everolimus indication to nonfunctional GI and lung NETs; first targeted therapy with efficacy across NET subtypes
NETTER-1 [9]	2017	^177^LU-DOTATATE + octreotide LAR 30 mg vs. octreotide LAR 60 mg	Progressive SSTR positive midgut NETs	PFS at 20 months; 65% vs. 11% (HR 0.18, 95% CI 0.11–0.29, *p* < 0.01)	ORR 18% with PRRT vs. 3% in control arm (*p* < 0.01); OS numerically higher with PRRT but not statistically significant	FDA approval of ^177^LU-DOTATATE, established PRRT as standard after progression on SSA in SSTR-positive disease
NETTER-2 [10]	2024	^177^LU-DOTATATE + octreotide LAR 30 mg vs. octreotide LAR 60 mg	Newly diagnosed G2 or G3 SSTR positive GEP-NETs	PFS; 23 vs. 9 months (HR 0.28, 95% CI 0.18–0.42, *p* < 0.01); 72% risk reduction	ORR 43% with PRRT vs. 9% in control arm (*p* < 0.01); DCR 91% vs. 68%	Supports first-line PRRT in higher grade well-differentiated NETs
ECOG-ACRIN E2211 [11]	2023	Capecitabine-temozolamide vs. temozolamide alone	Progressive pancreatic NETs	PFS; 23 vs. 14 months at interim analysis (HR 0.58, *p* = 0.02); 23 vs. 15 months at final analysis (HR 0.71, 95% CI 0.46–1.07)	MGMT deficiency associated with response	First randomized evidence for CAPTEM in pNETs, now preferred when tumor response needed for symptoms or cytoreduction
CABINET [12]	2025	Cabozantinib vs. placebo	Progressive well-differentiated GEP-NETs with ≥ 1 prior systemic therapy excluding SSA	PFS; 8 vs. 4 months in extrapancretatic NETs (HR 0.38, 95% CI 0.25–0.59, *p* < 0.01); 14 vs. 4 months in pNETs (HR 0.23, 95% CI 0.12–0.42, O < 0.01)	Better ORR in pNETs (18%) as compared to extra-pancreatic NETs (5%), many patients required dose reductions	FDA approval of cabozantinib for previously treated pNETs and extra-pancreatic NETs

Consensus guidelines help determine when LDT may be indicated or preferred amongst the aforementioned systemic therapies. The National Comprehensive Cancer Network (NCCN) guidelines on the Principles of Liver-Directed Therapy for NETLMs are widely used and were recently updated, as were the American Society of Clinical Oncology (ASCO) guidelines, while other consensus documents from the North American Society of Neuroendocrine Tumors (NANETs), European Society of Neuroendocrine Tumors (ENETs), and others are less current [24–29]. In general, all guidelines consider liver-dominant, unresectable NETLMs that are symptomatic, progressive, or causing bulk symptoms appropriate for LDT, but differ in when they recommend LDT in a patient’s treatment course and on recommendations regarding type of embolotherapy. In brief, per NCCN guidelines, embolotherapy is indicated in patients with NETLMs that are symptomatic or progressive on an SSA or other systemic therapy, or when immediate cytoreduction is warranted in those presenting with bulky disease, prior to failing systemic therapy. Both TAE and TACE are considered equivalent as “first-line” embolotherapies, while drug-eluding beads are not recommended due to increased hepatobiliary toxicity [30, 31]. TARE is recommended in certain scenarios, including lobar or segmental (as opposed to bilobar) disease distribution and in patients with prior bilioenteric anastomoses or biliary tract instrumentation, as there is less associated infectious risk with TARE as compared to TAE/TACE in this setting [32]. Bilobar TARE is not recommended early in disease courses due to the risk of radioembolization induced hepatotoxicity in long-term survivors. NCCN and ASCO guidelines incorporate LDTs early in the treatment course of patients with liver-dominant disease, whereas other guidelines integrate LDT at later stages. Looking ahead, the field eagerly awaits upcoming guidelines focused on surgical and liver-directed management of NETLMs resulting from a collaboration between NANETs, the Society of Surgical Oncology (SSO), the Americas Hepato-Pancreato-Biliary Association (AHPBA), and Society of Interventional Oncology (SIO). It should be noted that the evidence for liver-directed therapy in the treatment of NETLMs that informs such guidelines, and much of the recent data highlighted in the subsequent sections of this review, remains retrospective, single-center, and/or non-randomized in nature and are subject to selection bias amongst other biases. Published randomized trials comparing transarterial bland embolization (TAE), chemoembolization (TACE), and radioembolization (TARE) are currently lacking. Additionally, while guidelines aid in determining when liver-directed therapy is indicated and assist in choice of liver-directed therapy, ambiguity remains in scenarios where LDT, PRRT, and other systemic therapies may all be appropriate choices for a patient. While some studies integrating systemic and liver-directed options have been performed, and are discussed later on, there is an absence of comparative data. As a result, decisions to pursue LDT over an alternative systemic therapy, or combine or sequence such therapies in specific ways, frequently depend on anecdotal experience and multi-disciplinary decision-making.

### Solidifying TACE as a Repeatable Line of Therapy: The Gustave Roussy Experience

While the ideal embolotherapy for NETLM treatment remains debated, there is great value in rigorously studying a single embolic approach performed consistently over time. In this landmark study, Touloupas et al. described the long-term efficacy of TACE for NETLMs in 202 patients undergoing 555 TACE treatments over a 15-year period at Gustave Roussy, Europe’s largest cancer center [33]. Most patients (98%) had grade 1–2 NETs of the small bowel (39%), pancreas (26%), or lung (22%) and were followed for a mean of 8.2 years. TACE was employed early in patient’s treatment, with 74% having ≤ 1 systemic therapy beyond SSAs.

The authors conceptualized TACE as a repeatable line of therapy, rather than an isolated procedure - an increasingly important concept when considering LDT for NETLMS. They distinguished TACE sessions, defined as one procedure treating a portion of liver, from TACE cycles, which represent a set of TACE sessions that effectively treat all known NETLMs. Outcomes were assessed using time to liver progression (TTLP) after each TACE cycle and time to untreatable (unTACEable) progression (TTUP), defined as the time between first TACE and when TACE was no longer technically feasible or clinically recommended.

Among treated patients, 72% underwent one TACE cycle, 21% two cycles, and 7% three cycles. ORR and DCR were similar amongst the cycles, with ORR of 45% by RECIST, 66% by mRECIST, and DCR of 95%. While response did not differ between cycles, durability of response did, with TTLP declining from 20.5 months after the first cycle to 12.3 and 11.5 months after the second and third cycles, respectively (*p* = 0.024). Median TTUP was 26.2 months (95% CI 22.3–33.1), and was reached due to technical inability to perform further TACE in only 6%. Notably, > 25% of patients maintained disease control with TACE alone for more than 5 years, emphasizing the potential of LDT as a durable line of therapy. Median overall survival (OS) was 5.3 years (95% CI 4.1–6.7 years). Both OS and TTLP correlated with best imaging response after TACE, with mRECIST responders demonstrating a median OS of 80.5 months as compared to 39.6 months in non-responders (*p* < 0.001). Best mRECIST response was always seen at first imaging follow-up after each TACE cycle.

These findings reinforce TACE as an effective line of therapy for NETLMS. Rather than serving as an isolated intervention, TACE may function as a longitudinal treatment strategy capable of providing repeated disease control and delaying escalation to systemic therapy. That being said, this study must be interpreted within the limitations of its single-center, retrospective nature, and potential selection bias inherent to that. The applicability of these findings to other embolizations methods, including bland embolization and radioembolization, must also be considered.

### TACE Versus TAE: A Continuing Debate

Both TACE and TAE have been performed for treatment of NETLMs for decades, yet there remains little consensus on which modality provides the best outcomes or what factors should influence choice of embolotherapy. To date, TACE and TAE, as well as TARE, have demonstrated grossly similar effectiveness in symptom palliation and tumor control across a substantial body of primarily retrospective literature, with symptomatic response rates in the 85–90% range, radiologic response rates of 50–60%, and disease control rates of 80–90% [34–40]. Durability of response has been correlated more with tumor characteristics than embolization modality, with one study demonstrating a median hepatic PFS of approximately 18, 12, and 6 months in those with grade 1, 2, and 3 disease, respectively, without significant differences between embolotherapies [38, 39]. Recent randomized controlled trials and larger retrospective studies have sought to better differentiate between TACE and TAE, while other studies have evaluated whether different chemoembolic agents can offer improved outcomes.

The most anticipated trial in this area is the international multi-center Randomized Embolization Trial for NeuroEndocrine Tumor Metastases trial (RETNET trial; NCT02724540), which sought to compare TAE, TACE, and DEB-TACE in the treatment of NETLMs. While not yet published, preliminary results have been presented, and the trial has already influenced guidelines [41]. At first interim safety analysis, increased hepatobiliary toxicity identified in the DEB-TACE arm resulted in closure of this arm, and, in combination with other evidence, led to multiple guidelines recommending against DEB-TACE in NETLM treatment. The trial went on to enroll 151 patients with primarily grade 1–2 GEP-NETs (90%) in the TAE (*n* = 78) and TACE (*n* = 73) arms. Preliminarily, no significant difference in hepatic PFS (HR 1.40 [95% CI 0.80–2.46], *p* = 0.23) or overall PFS (HR 1.43 [95% CI 0.90–2.26], *p* = 0.13) was identified between TACE and TAE. Serious adverse events and toxicities occurred approximately 50% more frequently after TAE as compared to TACE. The final analysis of the RETNET trial will hopefully provide much needed insight into the decision between TACE and TAE for NETLMs, with expected publication in 2026–2027.

While awaiting prospective data, Ruff et al. recently published the largest single institution retrospective study comparing TACE and TAE utilizing propensity score matching (PSM) in a cohort of 412 patients, of which 83 underwent TAE and 329 underwent TACE [42]. The PSM cohort included 174 matched patients (TACE = 116, TAE = 58). No difference in complications was identified (TACE 23%, TAE 29%, *p* = 0.25), although TACE was associated with slightly worse post-procedural lab values. TACE was associated with a modest but statistically significant improvement in PFS of 12.1 ± 1.2 months compared to 10 ± 1.7 months for TAE (*p* = 0.002), although this did not translate into a difference in OS between embolotherapies.

The equivalence in outcomes between TACE and TAE raises questions as to whether the ideal chemoembolic agent is being used in TACE or if there are specific patient or tumor factors that should be considered in TACE vs. TAE decisions. A few studies have noted a better response to TACE as compared to TAE in pancreatic NETs, noting pNETs are considered more chemosensitive than small bowel NETs (sbNETs) [36, 43]. While most TACE regimens utilize some combination of doxorubicin, cisplatin, and/or mitomycin C, streptozosin (STZ)-based TACE has been proposed as a more NET-specific chemoembolization regimen, particularly for pNETs. Védie et al. recently compared 58 patients treated with STZ-TACE (43 pNETs, 15 sbNETs) to 58 treated with TAE (42 sbNETs, 16 pNETs) between 2006 and 2022 [44]. STZ-TACE demonstrated a higher ORR of 42% as compared to 22% for TAE (*p* = 0.045), although PFS was similar between both groups (12.9 vs. 12.1 months, *p* = 0.62). However, subgroup analysis demonstrated significantly longer PFS in pNETs treated with TACE as compared to TAE (12.9 vs. 6.4 months, *p* = 0.026), while no difference was observed in small bowel NETs (16.1 vs. 15.1 months, *p* = 0.47). Taken together, while STZ-TACE demonstrated improved ORR over TAE, this translated into a meaningful prolongation in PFS only in those with pNETs, supporting the concept that pNETs are relatively chemosensitive and may derive greater benefit from TACE as compared to sbNETs. A notable downside of STZ-TACE is the need to perform procedures under anesthesia due to the pain associated with STZ delivery, limiting widespread use.

Overall, the debate regarding whether TACE or TAE is more effective in NETLM treatment continues. Until a clear signal is seen, such decisions will continue to rely on sometimes conflicting, primarily retrospective data, differences in toxicity profiles, provider experience, and shared decision-making with patients.

### The Evolving Role of TARE

While TACE and TAE are considered first-line embolotherapy for NETLMs per published guidelines, emerging evidence and improvements in dosing methodologies are reshaping the role of TARE. Two prospective registries of y90 resin microsphere treatments recently reported NETLM outcomes. The CIRSE Registry for SIR-Spheres Therapy (CIRT) registry included 58 patients with NETLMs with a median OS of 33 months after TARE, the longest amongst metastatic cohorts [45]. Time from diagnosis to TARE was also longest in those with NETLMs, reflective of TAREs inclusion later in the disease course of such patients. Prognostic factors for OS were evaluated for the entire 1,027 patient registry, without NETLM sub-analysis, with worse outcomes noted in those with ascites, cirrhosis, worse ECOG performance status, more prior lines of therapy, and higher tumor burden. The radiation-emitting SIRspheres in non-resectable liver tumor (RESiN) registry separately reported on 170 patients with NETLMs [46]. OS was 33 months (95% CI: 25 months-not reached), similar to the CIRT registry, and 1-, 2-, and 3- year OS rates were 75%, 62%, and 46%, respectively. Median PFS was 25 months, with 1-, 2-, and 3-year PFS of 70%, 54%, and 35%, respectively. Prognostic factors for OS and PFS included worse ECOG performance status and ascites. The cohort was heavily pre-treated, with 42% having received chemotherapy, 33% prior embolotherapy, and 33% a targeted biologic agent.

While these registries demonstrated TARE’s effectiveness primarily in those undergoing treatment later in their disease courses, a recent multicenter retrospective study including 297 patients found that utilizing TARE earlier may yield better outcomes. Patients who underwent TARE as second-line therapy demonstrated a median hepatic PFS (hPFS) of 18.6 months as compared to 15.9 months when used in the salvage setting (*p* = 0.011) [47]. OS was also longer in the second-line setting as compared to the salvage setting, although this difference fell short of statistical significance (44.8 months vs. 30.6 months, *p* = 0.078). Both high tumor grade and hepatic tumor burden were associated with worse survival. While this study supports earlier use of TARE, this must be balanced with the potential for radioembolization induced chronic hepatotoxicity, which can limit patient’s future treatments options, and one must consider what role institutional preference or expertise played in embolotherapy decision making, as patients undergoing TARE may have also been candidates for TACE or TAE.

TARE treatment and dosing strategies that maximize tumor dose, thus optimizing response, and minimize dose to normal liver, mitigating toxicities, have been the focus of multiple recent studies. A brief account of dosing strategies will help contextualize this work. The historical models for dosing, including single compartment methods and the use of body surface area calculations, can result in underdosing, resulting in lack of tumor response, and overdosing, which may put patients at higher risk of injury to background liver. More contemporary multi-compartmental dosimetry models (partition model, voxel-based dosimetry) divide the liver into multiple tissue compartments (tumor, normal liver, etc.) rather than treating it as a single homogenous volume and incorporate patient-specific factors, such as tumor-to-normal liver uptake ratios, with the goal of maximizing y90 dose delivery to tumor while respecting dose limits to normal liver. The first NET-specific dose-response study utilizing the partition model, published by Chansanti et al. in 2017, evaluated 55 tumors across 15 patients treated with resin microspheres and found a tumor-absorbed dose of ≥ 193 Gy predicted response with high specificity, while a dose < 73 Gy predicted nonresponse with 100% specificity [48]. Ebbers et al. subsequently determined per-tumor dose-response in a cohort of 128 tumors in 26 patients treated with glass microspheres [49]. Median prescribed dose was 120 Gy (range 30–200 Gy) and, on a per-tumor basis, mean absorbed dose (MAD) was significantly related to response, with MAD of 170 Gy in responding tumors, 101 Gy in stable disease, and 67 Gy in progressive disease. A dose cutoff of 197 Gy (*p* < 0.01) for response was reported in another similar study, which also identified MAD, tumor grade, and tumor origin as predictors of PFS [50]. A recent study of resin microspheres found those with an objective response by mRECIST received a higher MAD to tumor of 107 Gy as compared to 70 Gy in those without response, and all tumors that received ≥ 120 Gy showed an objective response by mRECIST [51]. Utilization of such personalized dosimetry will help maximize TARE effectiveness and, taken together, these studies suggest a tumor MAD > 200 Gy would yield the most beneficial treatment response, although lower MADs of 120–150 Gy may be acceptable, bearing in mind target doses to tumor must be balanced with protection of normal background liver.

### Intra-Arterial PRRT Delivery: Time to Move on?

Optimization of PRRT delivery through intra-arterial infusion into the hepatic artery in patients with liver-dominant disease, with hopes of increasing uptake and absorbed dose within NETLMs via the “first pass” effect, has been described in a number of small studies. Retrospective and non-randomized prospective trials have demonstrated increased absorbed doses of ^177^Lu-DOTATATE within NETLMs after intra-arterial as compared to intravenous delivery [52, 53]. The largest retrospective series included 52 patients and demonstrated a DCR of 89% and ORR of 36%, with median PFS of 30 months and OS of 69 months [54]. A prospective trial of intra-arterial ^90^Y-DOTATOC failed to demonstrate increased NETLM uptake and was stopped early due to lack of efficacy. The only prospective randomized trial evaluating intra-arterial versus intravenous ^177^Lu-DOTATATE delivery is the LUTIA trial, in which 27 patients with bilobar NETLMs underwent four cycles of PRRT infused into the right or left hepatic lobe, with the contralateral lobe treated via a “second pass” effect and acting as the control [55]. The primary endpoint was tumor-to-non-tumor (T/N) uptake ratio in both lobes, and while an increased T/N uptake ratio was seen in the lobe treated intra-arterially, this difference was not significant and no difference in response was observed between the lobes at 3 or 6 months. Given the negative findings of this trial, it is unclear whether intra-arterial PRRT will continue to be pursued or abandoned.

### Combination Therapies: Looking for Synergy

Multiple studies have sought to combine therapies to exploit biologic synergy and improve outcomes in the treatment of NETLMs. When considering combining therapies, it is important to evaluate whether true synergy exists, meaning the combined outcome is better than either therapy could have achieved alone or if performed in stepwise fashion, and that cumulative toxicities are tolerable.

An early study in this area evaluated the combination of sunitinib with TAE, hypothesizing that VEGF inhibition after embolization would delay neoangiogenesis and prolong time to tumor progression [56]. The phase II trial demonstrated a 72% ORR and median PFS of 15 months while confirming a significant increase in circulating VEGF post embolization, and thus the rationale for the approach. However, patients found it difficult to tolerate sunitinib in the immediate post-embolization period, and many required dose reductions.

Subsequent studies have focused primarily on TARE in combination with various systemic therapies. The HEPAR PLuS single-center phase II clinical trial included 30 patients and evaluated the combination of PRRT with Holmium-166 radioembolization, which was performed within 20 weeks of the final PRRT treatment, with the goal of delivering an intensified treatment to liver metastases [57]. ORR at 3 months in the treated liver volume was the primary endpoint and reported at 43%. PFS and OS were not evaluated. One patient developed fatal radioembolization-induced liver disease, with other grade 3–4 toxicities including elevated liver enzymes (54%) and lymphopenia (23%). A subsequent propensity-score matched analysis comparing the HEPBAR PLuS patients to a historical PRRT cohort demonstrated a higher 2-year hPFS rate of 82% and best ORR of 71% with the combination treatment as compared to an hPFS of 50% and best ORR of 25% with PRRT alone [58]. However, no improvements in overall PFS or OS were identified. Mechanistically, this combination of PRRT and TARE more resembles an additive rather than synergistic approach, and these results demonstrate that while the regimen employed in the HEPAR PLuS study is relatively safe and feasible, any effect on hepatic PFS may not translate into an improvement in overall PFS or OS.

A phase 1b dose-escalation study evaluating the combination of pasireotide, everolimus, and y90 radioembolization was performed by Kim et al., hypothesizing the combination would optimize inhibition of the mTOR signaling pathway and potentiate the effects of TARE by inhibiting mTOR-mediated angiogenesis [59]. Twelve patients completed the treatment regimen, with TARE performed while patients were on the pasireotide and everolimus. Median PFS was 19 months and OS 46 months, and the regimen was well tolerated. The highest evaluated dose of 10 mg everolimus was recommended for further evaluation in a phase II and/or randomized trial to determine whether the combination was synergistic and improved outcomes compared to either treatment alone, neither of which have been performed.

**Fig. 1:**
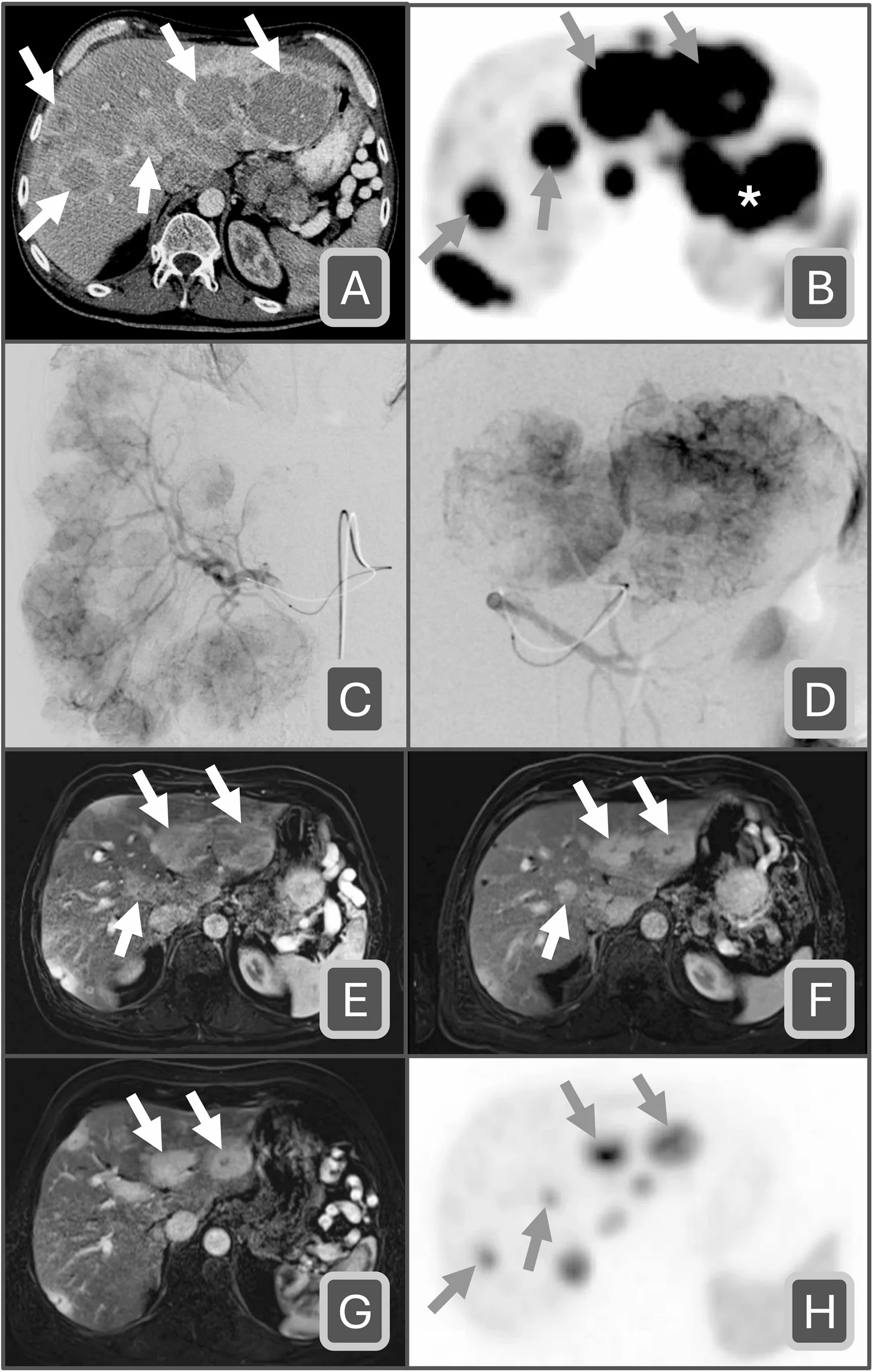
54 year-old with grade 2 metastatic pancreatic neuroendocrine tumor treated with the combination CAPTEM-y90 regimen. CAPTEM was administered for 12 cycles over 12 months, with right hepatic TARE performed with the second cycle and left hepatic TARE with the third cycle. (**A**) Pre-treatment arterial-phase CT demonstrates bulky bilobar liver metastases (white arrows). (**B**) Pre-treatment DOTATATE PET-CT redemonstrates the patient’s bulky bilobar liver metastases (gray arrows). The primary pancreatic tumor is also seen (white asterisk). (**C**) Right hepatic arteriogram performed during pre-y90 mapping demonstrates hypervascular right hepatic neuroendocrine tumor metastases. (**D**) Left hepatic arteriogram performed during pre-y90 mapping demonstrates hypervascular right hepatic neuroendocrine tumor metastases, corresponding to the bulky left side lesions seen on pre-procedural imaging. (**E**) 3-month follow-up subtracted early arterial phase MRI demonstrates a partial response in the treated NETLMs (white arrows) by both RECIST and mRECIST criteria. (**F**) 12-month follow up subtracted early arterial phase MRI demonstrates further decrease in size of the treated NETLMs (white arrows), with continued partial response. CAPTEM was stopped at this time. (**G**) 21-month follow up subtracted early arterial phase MRI demonstrates stability of treated NETLMs (white arrows). (**H**) 24-month follow-up DOTATATE PET-CT demonstrates significant shrinkage of NETLMs (gray arrows) compared to pre-treatment PET-CT (as demonstrated in figure part B), consistent with a sustained partial response throughout the 24 month follow-up period

The most promising data regarding synergistic treatments has been seen with the combination of capecitabine-temozolomide and radioembolization. Both drugs are radiosensitizers, providing rationale for synergy with and potentiation of the effects of radioembolization. After demonstrating safety, feasibility, and promising initial results in a small cohort of patients in 2018, long-term retrospective outcomes were reported in 37 patients with grade 2 NETs in 2024 [60, 61]. Mean duration of CAPTEM was 12 months, with pre-y90 mapping arteriography performed during the initial CAPTEM cycle, ensuring patient tolerance of the chemotherapy, and TARE performed on day 7 of subsequent CAPTEM cycles until all planned TARE sessions were completed, after which CAPTEM was continued until disease progression or intolerance. An ORR of 72% was seen in the treated NETLMs, with disease control rates of 100%. Median PFS was 36 months (95% CI 25–45 months) and median OS was 41 months (95% CI 24–87 months) from initiation of the regimen. These results exceeded historical outcomes with either therapy alone and suggested a synergistic treatment effect. Toxicities were no more than expected for either therapy alone, with thrombocytopenia, fatigue, and nausea being most common. An example of a patient treated with this regimen is demonstrated in Fig. 1. A multicenter phase II trial building on these results completed accrual of patients with grade 2 disease in 2025 and is now awaiting maturation of follow-up data, and a grade 3 disease arm was recently opened.

### Targeted Therapies for Solitary and Oligometastatic NETLMs

While NETLMs often present in a diffuse bilobar pattern, patients with solitary or limited hepatic metastases may benefit from focal therapies, such as ablation and radiation segmentectomy, which can provide durable local control while preserving liver parenchyma and maintaining future treatment options. NCCN guidelines recommend ablation for oligometastatic disease up to 4 lesions measuring < 3 cm, in line with studies demonstrating higher complete ablation rates with smaller lesions. Da Beare et al. reported complete responses in > 90% of those with lesions < 2 cm, 70–90% in those with lesions < 3 cm, and lower response rates for larger lesions [62]. In appropriate scenarios, percutaneous ablation of NETLMs has demonstrated similar local recurrence rates and OS compared to surgical resection, with lower complication rates and length of stay [63]. Recently, Matrood et al. described the use of ablation for local disease control with non-curative intent, focusing on ablation of oligometastatic hepatic progression in the setting of otherwise stable disease [64]. Ablation of isolated progressive lesions resulted in a median PFS of 63 weeks per procedure, extending to 69 weeks in those undergoing multiple procedures. The start of systemic therapy was delayed up to 123 weeks by utilizing ablation to control isolated progression. Ablation can also be combined with concurrent TACE to increase efficacy, especially in larger lesions (above 3 cm), and studies have demonstrated improved outcomes when combined, especially in patients grade 1 or 2 disease [65].

Location, proximity to adjacent critical structures, and other factors may preclude ablation of some solitary and oligometastatic NETLMs. Gordon et al. recently described radiation segmentectomy, in which higher “ablative” doses of y90 microspheres are delivered to 1 or 2 hepatic segments, as an alternative to ablation for such lesions [66]. In this study, 23 NETLMs not amenable to ablation or surgical resection were treated with a median dose of 235 Gy (95% CI 265–505 Gy) with ORR of 100% by mRECIST. Median overall PFS was 13 months, however, the median treated tumor TTP was not reached, suggesting that the treated lesions had durable response while patients progressed elsewhere. Overall, recent evidence suggests solitary, oligometastatic, or isolated progressive NETLMs can be well controlled by ablation, or radiation segmentectomy in those who aren’t ablation candidates, and such treatment can achieve high response rates.

### LDT in the Context of Recent Systemic Treatment Advances

As the role of liver-directed therapy evolves based on the aforementioned data, the evidence supporting new and existing systemic therapies continues to grow as well. As these options expand, decisions regarding liver-directed versus systemic therapy, particularly in those with both intra- and extra-hepatic metastases, become increasingly difficult. The lack of comparative data between parallel treatment options is the most significant hurdle to advancing the multidisciplinary care of NETLM patients. Current guidelines acknowledge this knowledge gap, stating there is “no data to support a specific sequence of regional versus systemic therapy” [24]. Two options in particular, first-line PRRT and later-line cabozantinib, have been advanced by the recent NETTER-2 and CABINET trials, respectively, and each warrants specific discussion of how they may influence practice, multidisciplinary decision-making, and the sequencing of LDT.

NETTER-2 randomized patients with newly diagnosed grade 2–3 GEP-NETs (Ki-67 10–55%) to first-line PRRT or high-dose SSA, demonstrating a markedly improved median PFS (22.8 vs. 8.5 months; HR 0.276) and ORR (43% vs. 9%) with PRRT [10]. While this established PRRT as a first-line option, the trial did not stratify by hepatic tumor burden or liver-dominant status, leaving unclear what role first-line PRRT should play in liver-only or liver-dominant disease - settings where early LDT has traditionally been considered. In the absence of comparative data, several practical considerations can help guide the integration and sequencing of PRRT and LDT. When liver-only or liver-dominant disease is symptomatic, bulky, rapidly progressive, or otherwise driving prognosis, LDT offers faster, more cytoreductive results and may be prioritized before PRRT. An embolization cycle can typically be completed in 2–3 months with an ORR of 80–90%, whereas a PRRT cycle takes roughly 8 months with a more gradual response and ORR of 30–40%. In such patients, beginning with LDT and reserving PRRT for when extra-hepatic disease drives progression, or when LDT is no longer effective or tolerated, is a reasonable strategy that preserves systemic options. Conversely, PRRT may be preferred when whole-body control is needed, hepatic tumor burden is low, or the patient cannot tolerate embolization. Cumulative hepatic radiation dose warrants specific attention when PRRT and radioembolization are sequenced, particularly as radiation-based therapies are utilized earlier in the disease courses in the post-NETTER-2 era. Although the HEPAR PLuS study showed no significant radiation-related toxicity and guidelines note “there is no evidence for or against the safety and sequencing of TARE and PRRT,” concerns about long-term hepatotoxicity remain [24, 57]. Baseline patient factors also matter. For example, bone marrow dysfunction or renal disease may favor LDT given the marrow and renal toxicities of PRRT, whereas hepatic dysfunction, patient frailty, or anatomic factors limiting embolization may favor PRRT.

While NETTER-2 focused on first-line therapeutic options, CABINET evaluated the multi-kinase inhibitor cabozantinib much later in patient’s disease courses, with most patients having received ≥ 2 prior systemic therapies beyond an SSA [12]. Patients were randomized to receive either cabozantinib or placebo, with cabozantinib demonstrating significantly improved PFS in both pancreatic (13.8 vs. 4.4 months) and extra-pancreatic (8.4 vs. 3.9 months) primaries and a disease control rate of 70–80%, supporting its use as a late-line treatment option. This heavily pre-treated population reflects patients who may be candidates for salvage LDT, or who have previously undergone LDT and are being considered for repeat treatment. CABINET did not report on prior LDT and, as with NETTER-2, did not stratify by hepatic tumor burden, so the influence of these factors is unknown. When weighing cabozantinib against LDT late in disease courses, the distribution and pace of disease again plays an important role in decision-making, as does patient tolerance of either therapy in this advanced-disease population. Regarding integration, the anti-angiogenic activity of cabozantinib, a result of VEGF-pathway inhibition, offers a theoretical rationale for combination with embolization; however, such agents impair wound healing and increase bleeding risk, and cabozantinib is typically held in the weeks before and after embolization. Whether combined or closely sequenced cabozantinib and embolization is safe or effective has not been studied.

**Fig. 2:**
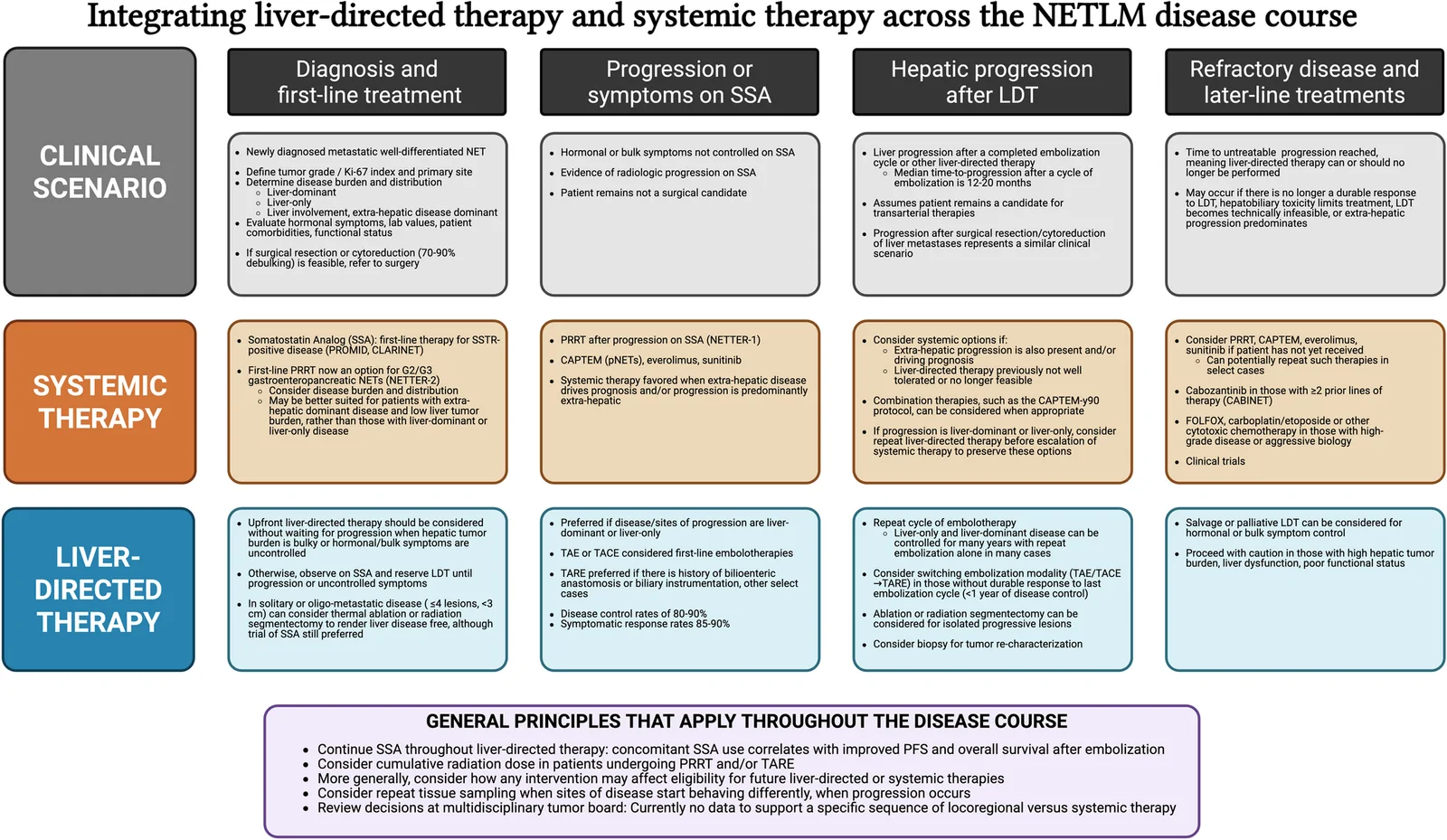
Example clinical scenarios within the disease course of neuroendocrine tumor liver metastases (NETLM) in which liver-directed therapies and systemic therapies may be considered. Practical considerations, guidelines recommendations, supporting studies, and general principles are included, noting individual cases should always be approached in a multidisciplinary fashion

Overall, the growing availability of effective systemic options, including first-line PRRT and later-line cabozantinib, does not diminish the role of LDT, but rather increases the importance of deliberate sequencing so that each therapy is deployed where it offers the greatest benefit. Example clinical scenarios of how LDT and systemic therapies may be integrated based on current evidence are provided in Fig. 2. Future studies evaluating the integration and sequencing of available therapies will be of utmost importance to NETLM patients.

### Emerging Technologies: Histotripsy for NETLMs

Histotripsy is a non-invasive technology that utilizes focused ultrasound to create cavitation bubbles that mechanically disrupt the target tissue within liver lesions. Following demonstration of safety and technical feasibility in the first-in-human THERESA trial and subsequent FDA clearance based on the multicenter HOPE4LIVER trial, the latter of which reported a 95% technical success, 7% major complication rate, and 83% complete response rate at 30 days in 49 treated tumors (2 of which were NETLMs), interest has grown regarding its potential role in the treatment of liver metastases [67, 68]. Liu et al. recently reported the first NETLM-specific experience in 27 patients, with intent for partial treatment of NETLMs for debulking in 93% and complete treatment of all known NETLMs in 7% [69]. Follow-up was available in a subset of patients and reported on a per-tumor basis. In 16 NETLMs with intent for complete treatment of that specific lesion, technical success was 69% and these tumors had a complete response throughout the follow-up period (median follow-up 120 days), while those without technical success demonstrated varying degrees of incomplete response or progression. In 19 NETLMs with intent for partial treatment, 53% demonstrated stable disease, 37% partial response and 10% a complete response at a median follow-up of 99 days. Major adverse events included acute kidney injury related to tumor histolysis in three patients (11%), potentially related to larger treatment volumes in these patients, and 1 death related to respiratory distress within a week of treatment. Overall, the role of histotripsy in the treatment of NETLMs will be better elucidated as evidence continues to build, but appears safe and most effective when complete coverage of tumors is achieved and treatment volumes are such that significant tumor histolysis is not induced.

### NETLM Biopsy: Increasingly Informing Treatment Decisions

There is a growing body of evidence demonstrating temporal and spatial heterogeneity of NETLMs, with Ki-67 index and tumor grade changing over time [70]. As the management of NETLMs becomes increasingly personalized, identifying such changes has implications for prognosis, treatment selection, clinical trial eligibility, and more. Concurrent biopsy at the time of LDT offers a unique and convenient opportunity to reassess tumor grade, obtain tissue for molecular profiling, and can be performed either at the time of ablation or embolization [71]. Early evidence regarding concurrent biopsy of NETLMs during LDT demonstrates both safety of the combined procedures and confirms that biopsy findings influence subsequent treatment decisions [72]. Recent literature also suggests molecular profiling may have implications for choice of transarterial therapy. The mutation and expression status of DAXX and ATRX, mutations frequently seen in pNETs, predicts response to LDT [73, 74]. Loss of DAXX/ATRX expression has been associated with significantly shorter PFS following ischemia-based therapies but increased radiation sensitivity and prolonged PFS following radioembolization, with median hPFS of 22 months after TARE as compared to 6 months in DAXX/ATRX wild-type tumors [73, 74]. As biologic and genetic insights begin to inform NETLM treatment plans more frequently, repeat and/or concurrent biopsy at the time of LDT should be considered and offered by IRs. Practically, image-guided biopsy can be easily integrated into ablation or embolization procedures, noting the addition of US-guided biopsy during embolization does not add significant procedural time. However, concurrent biopsy and embolization may carry a unique risk profile related to development of arterioportal or other fistulae in the immediate post-biopsy setting, although this infrequently affects subsequent embolization or requires additional intervention [72]. However, if biopsy information will be used to inform LDT decisions, it must be obtained ahead of time rather than at the time of embolization, thus necessitating a separate procedure. Depending on one’s institutional workflow, routine immunohistochemistry testing for mutations such as DAXX or ATRX may not typically be performed on biopsy samples, and NET tumor samples may not routinely be sent for molecular profiling, thus requiring IRs to work within their multidisciplinary NET treatment teams and with their pathology laboratories to establish NET-specific processes to obtain this increasingly important information.

### Future Directions

This review has highlighted numerous impactful recent studies, throughout which multiple themes have emerged that inform the most pressing needs for the field, primarily a continued need for high quality, prospective, randomized comparative data evaluating embolotherapy strategies and determining how best to integrate and sequence them with systemic therapies.

While the RETNET trial will provide the first randomized comparison of TAE and TACE, a similarly executed study comparing radioembolization to TAE or TACE for NETLMs will further inform embolotherapy selection, especially given the increasing sophistication of TARE dosimetry. Randomized data directly comparing these modalities and incorporation of prognostic factors such as grade, primary site, hepatic tumor burden, and others will help clarify which patients derive the greatest benefit from each approach. Regardless of choice of embolotherapy, there is a critical need for trials directly comparing or prospectively sequencing liver-directed therapies with systemic therapies, especially PRRT given its increasing use early in disease courses. Studies evaluating whether earlier LDT, systemic therapy, or defined combinations of these yield superior outcomes would greatly inform multidisciplinary treatment decisions. Potential synergies between LDT and systemic therapies should also be highlighted, as these are the areas in which integration of therapies provide the greatest patient benefit. Additional opportunities to guide therapeutic decisions may exist through incorporation of genetic and molecular data into LDT treatment decisions. As mentioned earlier, biomarkers such as DAXX/ATRX status predict differential response to embolotherapy and, through further validation, may enable individualized selection of embolotherapy. Further evaluation and prospective validation of such predictive markers and increased integration of concurrent biopsy and molecular profiling into LDT workflows will be essential in providing more personalized, biology-driven treatment decisions.

Additionally, the future of the field may include a number of new therapeutic interventions currently under evaluation. Ongoing trials regarding new chemotherapeutic options for TACE in NETLMs include a multi-center trial evaluating tirapazamine, a hypoxia-activated cytotoxic agent that may prove efficacious in the hypoxic environment created by concurrent embolization (NCT02174549). As data emerges for histotripsy, it may prove to be an additional option for select solitary or oligometastatic NETLMs. Given the limited number of open clinical trials for liver-directed therapy of NETLMs, the field is ripe for the study of innovative and targeted interventional therapies focused on this population.

## Conclusion

As summarized in Table 2, advances in embolotherapy approaches, publication of long-term outcome data, new treatment technologies, and identification of biologic predictors of response to liver-directed therapy are amongst the many recent developments that have refined the role of IRs within the multidisciplinary NET treatment team. As IRs continue to improve their ability to maximize disease control and preserve future treatment options through an evidence-based approach to selecting the right treatment at the right time for the right patient, they will play an increasingly important role in the treatment of NETLMs.

**Table 2:** Summary of emerging developments in liver-directed therapies for NETLMs

Topic	Previous evidence/paradigm	Recent findings	Clinical implications
TACE as a repeatable line of therapy	Limited evidence regarding outcomes and feasibility of repeat TACE for NETLMs	Long-term data demonstrating similar response and durable disease control across multiple TACE cycles, introduction of concepts such as time-to-untreatable progression [33]	Supports TACE as a repeatable therapy capable of providing long-term NETLM control and delaying escalation to additional systemic therapies in liver-dominant disease
TACE versus TAE	Choice of embolotherapy based on conflicting retrospective data, institutional preference	Preliminary RCT data from RETNET and recent propensity-matched analyses suggest overall similar outcomes; some studies suggest STZ-TACE advantage in pNETS [41, 42, 44]	Choice of TACE vs. TAE may not significantly affect outcomes with typical TACE regimens, emerging TACE regimens may improve outcomes in certain populations
Personalized dosimetry for TARE	Prior TARE dosing strategies inadequate, TARE as salvage therapy given concern for hepatotoxicity	Identification of tumor absorbed dose thresholds associated with response utilizing newer multi-compartment dosing, registry data and other studies suggest improved hPFS when TARE utilized earlier in disease course [47–51]	Improvements in TARE dosing may limit radioembolization-related hepatotoxicity while maximing dose to tumor, leading to better outcomes. Improved long-term safety would permit earlier use in NETLM treatment.
Radiation segmentectomy	Ablation and surgery were only local treatments for limited disease with published evidence	Recent studies demonstrate excellent local control with ablative-dose segmental radioembolization [66]	Expands evidence-based treatment options for lesions not amenable to surgery or thermal ablation
Intra-arterial PRRT	Intra-arterial infusion within hepatic artery hypothesized to improve uptake and outcomes	Randomized LUTIA trial failed to demonstrate meaningful clinical benefit compared to intravenous administration [55]	Best available evidence does not support routine use of intra-arterial PRRT
Combination liver-directed and systemic therapies	Therapies traditionally administered sequentially when separately indicated	Studies of TARE with PRRT, targeted therapies, and CAPTEM have demonstrated feasibility, safety, and efficacy. Synergistic effects seen with TARE and CAPTEM [61]	Synergistic treatment combinations can improve outcomes compared to either therapy alone, ongoing clinical trials will further evaluate the CAPTEM-y90 regimen
Emergence of histotripsy	No prior evidence for histotripsy in NETs	Early experiences demonstrate good local control when complete lesion coverage achieved, tumor histolysis and resulting kidney injury may occur with large treatment volumes [69]	Introduces a non-invasive and non-thermal treatment option, exact role in NETLM treatment will require additional studies
Genetic predictors of response	No consideration of tumor genetics when determining embolization modality	DAXX/ATRX mutation status in pNETs correlates with outcome of ischemia-based and radiation-based embolotherapies [73, 74]	Biologically-informed liver-direct therapy selection and personalized treatment strategies may become more commonplace

## Key References

- Touloupas C, Faron M, Hadoux J, Deschamps F, Roux C, Ronot M, Yevich S, Joskin J, Gelli M, Barbé R, Lamartina L. Long term efficacy and assessment of tumor response of transarterial chemoembolization in neuroendocrine liver metastases: a 15-year monocentric experience. Cancers. 2021 Oct 26;13(21):5366.
  - ○ This study describes a long-term single-center experience with TACE for NETLMs, defines a number of terms useful in considering TACE as a repeatable line of therapy, and provides evidence for the effectiveness of repeat TACE.
- Ebbers SC, Barentsz MW, de Vries-Huizing DM, Versleijen MW, Klompenhouwer EG, Tesselaar ME, Stokkel MP, Brabander T, Hofland J, Moelker A, van Leeuwaarde RS. Intra-arterial peptide-receptor radionuclide therapy for neuro-endocrine tumour liver metastases: an in-patient randomised controlled trial (LUTIA). European Journal of Nuclear Medicine and Molecular Imaging. 2024 Mar;51(4):1121-32.
  - ○ The LUTIA trial is the first and only randomized controlled trial evaluating intra-arterial PRRT and found no clinically significant benefit to this approach, challenging the rationale for hepatic arterial delivery.
- Braat AJ, Bruijnen RC, van Rooij R, Braat MN, Wessels FJ, van Leeuwaarde RS, van Treijen MJ, de Herder WW, Hofland J, Tesselaar ME, de Jong HW. Additional holmium-166 radioembolisation after lutetium-177-dotatate in patients with neuroendocrine tumour liver metastases (HEPAR PLuS): a single-centre, single-arm, open-label, phase 2 study. The Lancet Oncology. 2020 Apr 1;21(4):561-70.
  - ○ HEPAR PLuS was the first prospective study to combine PRRT with radioembolization and demonstrated acceptable toxicities, with response rates of approximately 40%.
- Soulen MC, Teitelbaum UR, Mick R, Eads J, Mondschein JI, Dagli M, van Houten D, Damjanov N, Schneider C, Cengel K, Metz DC. Integrated capecitabine–temozolomide with radioembolization for liver-dominant G2 NETs: long-term outcomes of a single-institution retrospective study. Cardiovascular and Interventional Radiology. 2024 Jan;47(1):60-8.
  - ○ This study demonstrated that the combination of capecitabine-temozolomide with Y-90 radioembolization could achieve disease control rates and progression-free survival exceeding that expected of either therapy alone, supporting treatment synergy, which is now being studied in a multi-center prospective trial.
- Gordon AC, Savoor R, Kircher SM, Kalyan A, Benson III AB, Hohlastos E, Desai KR, Sato K, Salem R, Lewandowski RJ. Yttrium-90 radiation segmentectomy for treatment of neuroendocrine liver metastases. Journal of Vascular and Interventional Radiology. 2025 Feb 1;36(2):293-300.
  - ○ This is the first study of radiation segmentectomy for NETLMs and demonstrated excellent local control with minimal toxicity, supporting ablative-dose Y-90 as a treatment option for solitary or oligometastatic NETLMs.
- Liu E, von Breitenbuch P, Kemp A, Mauceri H, Whitely P, Burns J, Sanchez D, Ziemlewicz TJ. Histotripsy for neuroendocrine liver metastases: Early single-institution outcomes and safety. Surgery. 2026 Jun 1;194:110084.
  - ○ This study provided the first dedicated evidence for histotripsy of NETLMs and helps guide future consideration of this emerging technology in this patient population.
- Solivio C, Yuan G, Rizzo AJ, Graham MK, Shenker L, Vista W, Gil A, Singh H, Alexander E, Gonzalez-Aguirre A, Latzman J. Loss of DAXX/ATRX Protein Expression Results in Ischemia Resistance and Radiation Sensitivity in Pancreatic Neuroendocrine Tumor Cells and Is Associated with Improved Response to Trans-Arterial Radioembolization. Neuroendocrinology. 2025 Sep 22;115(9):730-40.
  - ○ This study linked tumor genomics to liver-directed therapy response, demonstrating that DAXX/ATRX-deficient pNETs may preferentially benefit from radioembolization over ischemia-based embolization treatments.

## Data Availability

No datasets were generated or analysed during the current study.

## Abbreviations

NET: Neuroendocrine tumor
NETLM: Neuroendocrine tumor liver metastases
LDT: Liver-directed therapy
IRs: Interventional Radiologists
PRRT: Peptide receptor radionuclide therapy
TAE: Transarterial bland embolization
TACE: Transarterial chemoembolization
TARE: Transarterial radioembolization
SSA: Somatostatin analog
GEP-NET: Gastroenteropancreatic neuroendocrine tumor
pNET: Pancreatic neuroendocrine tumor
DCR: Disease control rate
ORR: Objective response rate
PFS: Progression-free survival
OS: Overall survival
hPFS: Hepatic progression-free survival
MAD: Mean absorbed dose
